# Vitamin D status and its influence on outcomes following major burn injury and critical illness

**DOI:** 10.1186/s41038-018-0113-4

**Published:** 2018-04-16

**Authors:** Khaled Al-Tarrah, Martin Hewison, Naiem Moiemen, Janet M. Lord

**Affiliations:** 1 Institute of Inflammation and Ageing, Birmingham University Medical School, Birmingham, B15 2TT UK; 2 Scar Free Foundation Centre for Burns Research, University Hospital Birmingham Foundation Trust, Birmingham, B15 2WB UK; 3 Institute of Metabolism and Systems Research and Centre for Endocrinology Diabetes and Metabolism, The University of Birmingham, Birmingham, B15 2TT UK

**Keywords:** Vitamin D, Critical care, Burns, Trauma, Thermal injury, Critically ill

## Abstract

Vitamin D deficiency is common among the general population. It is also observed in up to 76% of critically ill patients. Despite the high prevalence of hypovitaminosis D in critical illness, vitamin D is often overlooked by medical staff as the clinical implications and consequences of vitamin D deficiency in acute contexts remain to be fully understood. Vitamin D has a broad range of pleotropic effects on various processes and systems including the immune-inflammatory response. 1α,25-dihydroxyvitamin D (1,25(OH)_2_D), has been shown to promote a tolerogenic immune response limiting deleterious inflammatory effects, modulation of the innate immune system, and enhancement of anti-microbial peptides. Vitamin D deficiency is frequently observed in critically ill patients and has been related to extrinsic causes (i.e., limited sunlight exposure), magnitude of injury/illness, or the treatment started by medical doctors including fluid resuscitation. Low levels of vitamin D in critically ill patients have been associated with sepsis, organ failure, and mortality. Despite this, there are subpopulations of critical illness, such as burn patients, where the literature regarding vitamin D status and its influence on outcomes remain insufficient. Thermal injury results in damage to both burned and non-burned tissues, as well as induces an exaggerated and persistent immune-inflammatory and hypermetabolic response. In this review, we propose potential mechanisms in which burn injury affects the vitamin D status and summarizes current literature investigating the influence of vitamin D status on outcomes. In addition, we reviewed the literature and trials investigating vitamin D supplementation in critically ill patients and discuss the therapeutic potential of vitamin D supplementation in burn and critically ill patients. We also highlight current limitations of studies that have investigated vitamin D status and supplementation in critical illness. Thermal injury influences vitamin D status. More studies investigating vitamin D depletion in burn patients and its influence on prognosis, via standardized methodology, are required to reach definitive conclusions and influence clinical practice.

## Background

Vitamin D insufficiency and deficiency is common in the general population [1] and can be present at up to 76% in critically ill patients [2]. This is concerning as vitamin D is increasingly recognized for its wide-ranging biological effects, including modulation of bone metabolism and muscle mass, enhancing immune function and cardiovascular effects [3, 4]. Despite these roles, the clinical implications of hypovitaminosis D remain partially understood and therefore often overlooked in acute clinical contexts including burns and trauma. The literature investigating vitamin D deficiency and its consequences in adult burn patients is limited. Following thermal injury, patients are at prone to develop low vitamin D levels, the impact on short and long-term outcomes of which are relatively unknown.

This review aims to discuss current understanding of vitamin D and its role in critically ill patients, the effect of burn injury on vitamin D status, the influence of vitamin D levels on outcomes in burn patients, the therapeutic potential of vitamin D, and current knowledge gaps.

## Review

### Overview of vitamin D axis

**Fig. 1:**
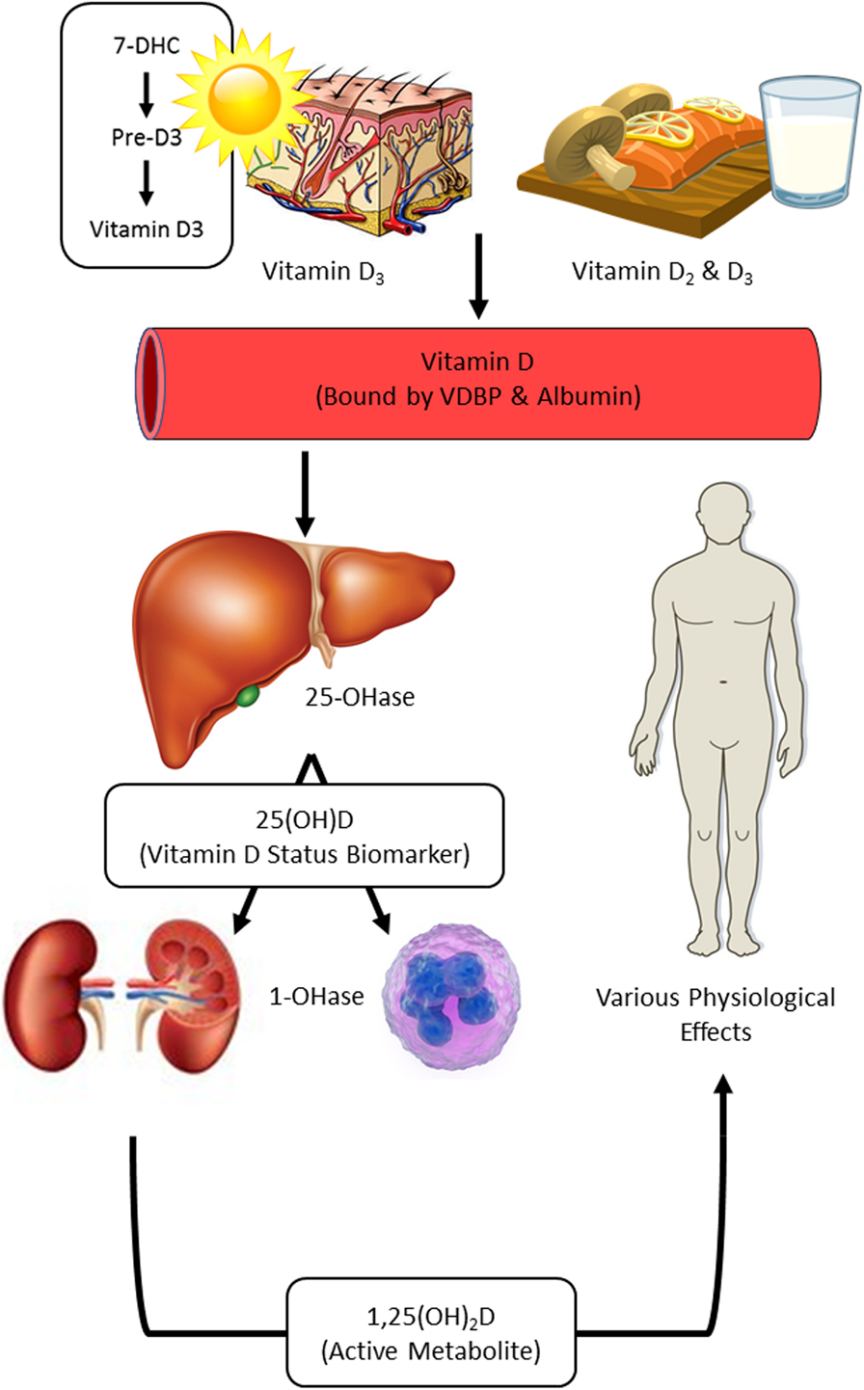
Overview of the vitamin D axis

Sunlight and nutrition, including dietary supplementation, are the main sources of vitamin D in humans. Solar ultraviolet B radiation infiltrates the skin converting 7-dehydrocholesterol (7-DHC) to pre-vitamin D_3_ (pre-D_3_), which is subsequently converted to vitamin D_3_ [4–6]. Vitamin D (D_2_ and D_3_) is also found naturally in certain foods (oily fish, mushrooms, egg yolk) and fortified food products, including cereals, cheese, and milk [4, 5]. Vitamin D from the skin and diet is then transported to the liver bound to vitamin D-binding protein (VDBP) and albumin, where it is hydroxylated to 25-hydroxyvitamin D (25(OH)D) [4–6]. This is used to determine a patient’s vitamin D status. 25(OH)D is then metabolized by the enzyme 25-hydroxyvitamin D-1αhydroxylase (CYP27B1) in the kidneys to the active form of vitamin D, 1α,25-dihydroxyvitamin D (1,25(OH)_2_D) [4–6], which is then transported to various target cells and tissues where it interacts with intracellular vitamin D receptors (VDRs) to exert transcriptional effects. In addition to the kidneys, various extra-renal sites (such as macrophages) are reported to contain CYP27B1 permitting direct metabolism of 25(OH)D to exert pleotropic effects via autocrine means [7] (Fig. 1).

### Overview of vitamin D biological effects

Classically, vitamin D is associated with musculoskeletal health by maintaining calcium homeostasis and bone mineralization, decreasing the risk of muscle weakness, osteopenia, osteoporosis, and fractures [4, 7]. Vitamin D exerts most of its physiological effects via 1,25(OH)_2_D which when bound to its cognate nuclear VDR is able to act as a transcription factor in concert with its retinoid X receptor heterodimer partner [8, 9]. Gene expression analysis of 53 different tissues from over 500 human donors has shown VDR gene expression in more than half of samples including the adipose tissue, adrenal glands, bladder, colon, fibroblasts, kidney, liver, lung, lymphocytes, pituitary glands, and skin [10, 11]. Accordingly, vitamin D actions are not limited to the skeletal system. The effects of vitamin D on various cell types and tissues are summarized in Table 1.

**Table 1:** Effects of vitamin D on various human cell types and tissues

Target cells/tissues	Effects of vitamin D	Reference
Adipocytes	• Inhibits intracellular fat accumulation • Enhances basal lipolysis without cell toxicity • Upregulation of β-oxidation-related genes, lipolytic enzymes, and vitamin D-responsive genes • Increased levels of nicotinamide adenine dinucleotide and sirtulin 1 expression	[105]
Cardiomyocytes	• Inhibition of cell proliferation without apoptosis • Downregulation of expression of genes associated with cell cycle regulation • Promotes cardiomyotube formation • Induces cardiac differentiation	[106, 107]
Hepatocytes	• Protects against insulin resistance • Downregulates fibrogenic TGF-β signaling • Anti-inflammatory effects by inhibiting monocyte activation and TNF-α and IL-1 expression	[108–110]
Myocytes	• Modulation of calcium homeostasis and influx • Induces cellular proliferation and differentiation • Protects against insulin resistance • Stimulation of arachidonic acid mobilization	[111, 112]
Nephrocytes	• Upregulation of cellular metabolic activity, IL-6, and reactive oxygen species • Restoration of transepithelial barrier function	[113]
Neurons	• Neuroactive steroid modulating spontaneous regular firing, actin potential duration, and intrinsic excitability • Enhances sensitivity to neurotransmitters and neurotransmitter receptors • Upregulation of neuronal growth factors, neurotrophin 3, and glial cell line-derived neurotrophic factor	[114, 115]
T cells	• Inhibits Th1/Th17 chemokine/cytokine secretion (CXCL-10, IFN-γ, TNF-α, and IL-17) • Enhances Th2 cytokine release (IL-4 and IL-5)	[17, 116]
B cells	• Downregulates the proliferation of memory B cells • Inhibits plasma cell differentiation • Reduces Ig production	[117]
Antigen-presenting cells	• Inhibits the expression of class II MHC molecules (HLA-DR) • Inhibition of co-stimulating molecule expression (CD80, CD83, and CD86) • Augments chemotaxis and phagocytosis of monocytes • Downregulates the maturation of dendritic cells • Induces tolerogenic dendritic cells capable of inducing Treg cells • Inhibits IL-12 p70 release • Decreases macrophage-stimulated pro-inflammatory cytokine production (IL-1, IL-1β, IL-6, IL-8, MCP-1, and RANTES)	[118–121]
NK cells	• Inhibition of NK cell development and differentiation • Reduced INF-γ and cytotoxicity	[122]

*TGF-β* Transforming growth factor-β, *TNF-α* Tumor necrosis factor, *IL* Interleukin, *IFN-γ* Interferon-γ, MCP-1 Monocyte chemotactic protein 1, *NK* Natural killer

Of relevance to this review, vitamin D has a broad range of beneficial effects on the immune system [12]. An association between the adaptive immune system and vitamin D status was initially observed when VDR levels were shown to be enhanced in activated T and B cells [13]. In VDR-expressing T cells, 1,25(OH)_2_D promotes a tolerogenic immune response by favoring Th2 and Treg cell differentiation over the more inflammatory Th1 and Th17 cells, thereby limiting deleterious inflammatory activity [14–18]. Other immune-modulatory effects of vitamin D include differential modulation of the response of the innate immune system (monocytes, macrophages and dendritic cells) [19] with upregulation of anti-microbial peptides such as cathelicidin and β-defensin 2 from various cells including human keratinocytes and intestinal epithelial cells [20, 21], enhancement of autophagy of intracellular microbes [22], and regulation of antigen-presentation in dendritic cells, monocytes and macrophages to facilitate a non-exaggerated immune response [23]. Crucially, antigen-presenting cells from the innate immune system express the vitamin D-activating enzyme CYP27B1 and are therefore able to metabolize 25(OH)D in a tissue-specific fashion [24]. This “intracrine” mode of 25(OH)D metabolism appears to be the principal mechanism by which vitamin D is able to regulate T cell function [25] and provides a mechanism by which vitamin D deficiency (low serum 25(OH)D) can influence immune function. The various effects of vitamin D upon the immune response are summarized in Table 1.

DBP and albumin are the main transporters of vitamin D. However, sterol-binding capacity is not the only attribute of DBP and albumin. Multiple roles of DBP have been described including actin scavenging, binding of fatty acids and endotoxins, modulation of immune and innate immune responses, and influence on bone metabolism via DBP-macrophage activating factor [26]. Albumin has been reported to exert antioxidant, immune-modulatory, and anti-inflammatory effects, as well as antibiotic transportation and endothelial stabilization [27, 28].

### Vitamin D in critically ill and trauma patients

Considering the pleotropic effects of vitamin D, its role in the severely ill has been a subject of growing interest. Thousands of patients are admitted to intensive care units (ICUs) each year [29], and up to 77% of critically ill patients have vitamin D deficiency [2, 30–33]. Alizadeh et al. reported that 74% of critically ill surgical patients exhibited vitamin D deficiency [34]. Similarly, Dickerson et al. reported that 76% of critically ill patients following traumatic injury were vitamin D deficient or severely deficient [2]. In such contexts, it is important to recognize patient demographic factors that may be associated with vitamin D deficiency including age, ethnic group (skin pigmentation), obesity, medical history (such as malabsorption pathologies and liver/renal disease), season, latitude, and time of day [4]. However, it is also vital to comprehend that vitamin D deficiency may itself be a consequence of illness.

Low serum 25(OH)D levels has shown a significant association with the magnitude of the critical illness and systemic inflammatory response syndrome (SIRS) [33, 35, 36]. The well-documented immunomodulatory effects of 1,25(OH)_2_D suggest that vitamin D deficiency may be a causative factor for critical illness and resulting morbidity and mortality. The observed vitamin D deficiency in critically ill and trauma patients may be due to diminished epidermal vitamin D production secondary to limited sunlight exposure and malnutrition, as well as enhanced conversion of 25(OH)D to active 1,25(OH)_2_D to meet increased tissue demand, notably to promote 1,25(OH)2D-mediated immunoregulatory effects [37]. Finally, critical illness, notably in the setting of inflammation, may promote enhanced catabolism of 25(OH)D and 1,25(OH)_2_D to downstream metabolites via the enzyme 24-hydroxylase (CYP24A1) [38]. Interpretation of circulating vitamin D levels in critical illness is further complicated by the fact that critically ill patients usually require major fluid resuscitation resulting in low levels of 25(OH)D and 1,25(OH)_2_D secondary to acute fluid shifts and hemodilution [39]. Vitamin D concentration in critically ill patients post-resuscitation may take, at least, a few days to recover [39]. Secondly, VDBP and albumin levels fall, as part of the systemic inflammatory response, reducing plasma levels of 25(OH)D significantly [40–42]. This appears to be the case in the acute phase of the response to injury. Furthermore, disruption of the vitamin D axis in ICU patients can be attributed to hepatic, parathyroid, and renal dysfunction, as well as reduced end organ resistance [43].

Clinical studies have associated low levels of circulating vitamin D with various poor outcomes in critically ill patients including sepsis [44, 45], organ failure [46, 47], and short- and long-term mortality [30, 44, 46, 48, 49]. Similar findings have also been reported among both critically ill surgical or trauma patients. For example, low vitamin D levels correlated with higher infection rates, length of stay, duration of organ dysfunction, ICU readmission, surgical intensive care treatment costs, and mortality [50–53]. However, such associations are not universal; other observational studies have reported no association between vitamin D deficiency, sepsis, and mortality [54, 55], as well as other ICU outcomes such as duration of ventilation and length of stay [56]. Despite this, several meta-analysis studies have concluded that vitamin D deficiency is associated with significantly increased susceptibility to infections and sepsis, as well as greater incidence of mortality in critically ill patients [57–59].

### Burn injury pathophysiology and its impact on vitamin D levels

Critically ill populations are clearly identified to be at risk of vitamin D deficiency, but there are sub-populations where there is insufficient literature on the status of vitamin D and its influence on outcomes, including patients with thermal injuries. Although studies investigating vitamin D levels in burns patients are scarce, vitamin D levels have been shown to decrease following thermal injury [60, 61]. This may be both as a primary effect of the injury or secondary response to the injury itself and/or the clinical management initiated such as fluid resuscitation and use of pressure garments.

Burn is a severe form of injury associated with a marked pathophysiological immune-inflammatory response. Thermal injury induces a unique “genomic storm” altering 80% of the leukocyte transcriptome leading to prolonged simultaneous and rapid stimulation of innate (both pro- and anti-inflammatory genes) and suppression of adaptive immune responses [62]. Clinical studies have recently characterized the immune response following burn injury demonstrating prolonged neutrophil dysfunction and release of immature granulocytes lasting up to 28 days [63], reduced numbers and impaired expression of CD14 +/HLA-DR + monocytes persisting up to 30 days [64], and downregulation of NKG2D (a natural cytotoxicity receptor in natural killer cells) ligands resulting in immunosuppression [65]. The presence of concurrent upregulation of granulocyte-macrophage colony-stimulating factor (GM-CSF), interleukin (IL)-10, and other cytokines following injury indicates an over-compensating response by the body [66], which may last for up to 3 years post-burn [67].

Thermal injury is associated with a hypermetabolic response. The metabolic changes in burn injury and other forms of trauma are similar but differ in magnitude and persistence. These changes are characterized as a two-phase response: the “ebb” phase within 48 h where metabolism, cardiac output, and oxygen consumption are reduced, followed by the “flow” phase at approximately 120 h post-injury where these parameters increase and plateau [68]. This metabolic response involves peripheral lipolysis and free fatty acid [69] oxidations leading to acute, global, and complex increase in serum FFA levels [70]; browning of subcutaneous white adipose tissue [71]; systemic induction of endoplasmic reticulum (ER) stress and unfolded protein response [72]; and up to a sixfold increase in breakdown rates of skeletal muscle protein [73]. It is estimated that severe burns cause a surge of resting energy expenditure up to 140% [74], which can be prolonged [67]. This persistent hypercatabolic, hyperinflammatory response leads to wasting of the patient’s tissues and organ, ultimately leading to poorer outcomes.

In addition, fluid and proteins are translocated into burned and non-burned tissues leading to hypovolemia, damaging tissues through direct and indirect means [75]. The extent of which is reflected by elevated levels of damage-associated molecular patterns (DAMPs) such as high-mobility group box protein 1 following burn injury [76, 77]. There is also disruption of local and systemic vascular permeability via the margination of immune cells including neutrophils, macrophages, and lymphocytes, as well as various inflammatory mediators [78]. This ultimately leads to an instant shift of intravascular fluid to the interstitial space. To address this issue and circumvent the consequences of hypo/hypervolemia, multiple formulations have been established throughout burn care history to optimize fluid resuscitation [79].

Severe burn injury thus induces persistent disturbances of multiple immuno-inflammatory and physiological responses simultaneously, befitting the designation: persistent inflammation, immunosuppression, and catabolism syndrome, PICS [80]. This includes reduced circulating levels of vitamin D and its carrier proteins, VDBP and albumin [61]. Based on literature concerning non-burn and burn trauma, we propose that this can be explained through a variety of potential mechanisms: First, as previously postulated [37], there may be attempts to maintain immune homeostasis via increased conversion of 25(OH)D to active 1,25(OH)_2_D, thereby reducing circulating 25(OH)D levels. In individuals who are vitamin D-sufficient prior to injury, this effect may have a negligible impact on serum 25(OH)D status, but for those who are vitamin D-deficient at injury, this effect may lead to exacerbation of low serum 25(OH)D concentrations; second, fluid resuscitation and a compromised vascular integrity results in decreased serum levels of vitamin D and its metabolites secondary to hemodilution and fluid shifts [39]. VDBP and albumin are also affected in the acute response, thereby reducing bound vitamin D levels and impacting its delivery to target tissues [40–42]. This phenomenon of binding protein extravasation would be temporary as microvascular integrity is re-established 6 h following thermal injury [81]. Third, VDBP levels are reduced in the acute stage buffering actin’s deleterious effects as part of the actin scavenging system [69, 82].

Although unknown, it appears that VDBP levels recover during the acute phase of thermal injury [83], while albumin levels may recover as early as 6 months [84]. Due to this multiplicity of factors, interpretation of 25(OH)D levels and diagnosing vitamin D deficiency in burn patients remain challenging [85].

**Fig. 2:**
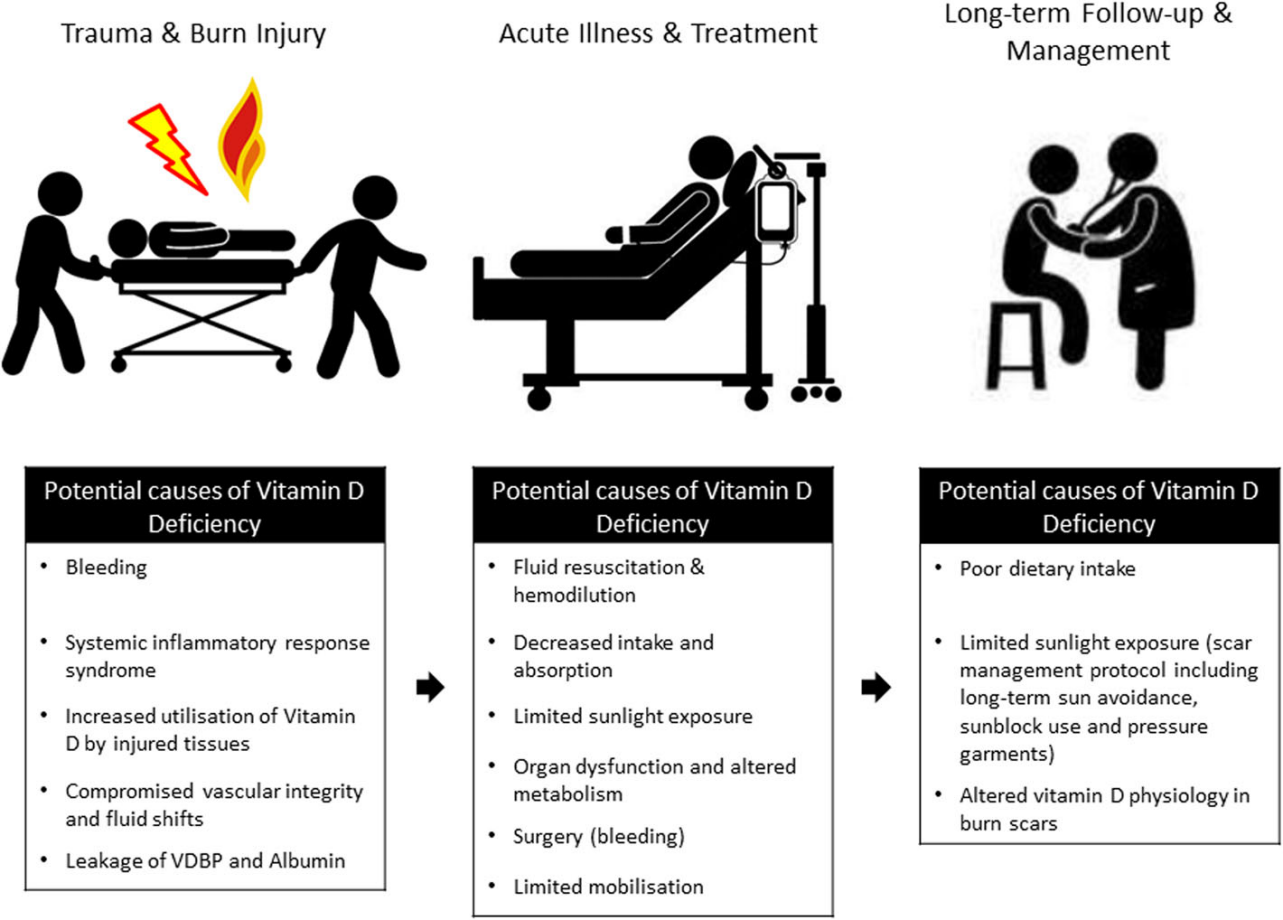
Potential causes of vitamin D deficiency following injury

Vitamin D levels following severe thermal injury can also be reduced secondary to extrinsic causes including prolonged in-hospital stay (including ICU), prolonged immobilization, and lack of supplementation. Although critically ill burn patients receive oral or enteral feed supplements, current regimens have proved ineffective in replenishing vitamin D levels in the acute phase [60]. Furthermore, current long-term burn management regimens involve scar management comprising mainly of sun avoidance and protection, as well as the use of pressure garments. These factors minimize sun exposure, hence reducing 25(OH)D levels. In addition, both burn scar and adjacent normal skin in burn patients exhibit subnormal conversion levels of 7-DHC to pre-D_3_ compared to healthy individuals [86]. This further potentiates vitamin D deficiency, resulting in low levels of 25(OH)D and 1,25(OH)2D for many years, at least seven, following burn injury [87]. The potential causes of hypovitaminosis D following injury are summarized in Fig. 2.

### Influence of vitamin D status on outcomes in burn patients

Low vitamin D levels in patients with minor burns, median TBSA of 5%, have been associated with increased length of hospital stay [88]. Although not statistically significant, the authors also observed higher complication rates in burn patients with low vitamin D including sepsis, pneumonia, cardiovascular complications, and graft loss [88]. It is important to note that low vitamin D status in this study cohort most likely represents the population’s pre-injury 25(OH)D levels rather than a consequence of the burn. Furthermore, this study has some limitations. Thirty percent of the cohort was admitted to the ICU with relatively minor injuries, which is unusual. No description of patient pre-morbid state or other pathologies were reported which may potentially affect vitamin D status or outcomes in general. In addition, the cohort is comprised mostly of minor partial thickness thermal injuries limiting its application in severe burns. There are no reports investigating the influence vitamin D levels on short-term outcomes of major burn patients.

Low serum levels of vitamin D in major burn patients have shown to persist for at least 1 year [83]. Long-term outcomes assessed include bone mineral density and leg muscle strength. Although not statistically significant, quadriceps muscle strength was lower at month 12 than the month of injury in burn patients with low vitamin D levels [83]. Another long-term consequence of major thermal injury is scarring. A cross-sectional study has reported a strong negative correlation between circulating 25(OH)D levels at year 1 post-injury and subjective scar measures (modified Vancouver scar scale) [89]. Both studies were limited by small sample sizes. There are no other studies investigating the influence of vitamin D on long-term outcomes of burned adult patients.

Studies have reported increased incidence of long bone fractures among children with major burns following discharge [90]. This is most likely attributed to reduced bone mineral density and vitamin D levels [87, 91]. Vitamin D supplementation in pediatric burn patients may be beneficial in reducing fracture risk [91].

### Therapeutic potential of vitamin D supplementation

Vitamin D_3_ supplementation may be associated with decreased mortality in the general population [92]. In addition, vitamin D status has been associated with adverse outcomes in the critically ill. Despite this, there are only a few clinical studies that have evaluated vitamin D supplementation in critically ill patients. In 2014, Amrein et al. conducted the largest randomized controlled trial (RCT) to date investigating the influence of a high-dose bolus enteral vitamin D3 supplementation on outcomes of 475 critically ill medical and surgical adult patients with vitamin D deficiency (≤ 20 ng/mL), the VITdAL-ICU trial [93]. The authors concluded that high-dose vitamin D3 did not reduce hospital length of stay, hospital mortality, or 6-month mortality [93]. However, they observed lower hospital mortality following subgroup analysis of patients with severe vitamin D deficiency (≤ 12 ng/mL) at baseline [93]. A systematic review and meta-analysis of 7 RCTs (716 patients) concluded that vitamin D administration was associated with decreased mortality in critically ill patients without serious adverse events [94]. Interestingly, another recent meta-analysis of 6 RCTs (695 patients) reported no improvement on outcomes in critically ill patients supplemented with vitamin D [95]. The difference between these two studies is related to inclusion and exclusion of various trials in the analysis. Other potentially important confounders in both studies are the inclusion of trials investigating cholecalciferol (vitamin D) and calcitriol (1,25(OH)_2_D) using various dosing regimens that were administered through different routes (enteral and intravenous). Furthermore, the VITdAL-ICU trial has a larger cohort than all the other RCTs combined and therefore has major influence in the statistical analysis. These trials are summarized in Table 2.

**Table 2:** Summary of clinical trials investigating vitamin D supplementation in critically ill patients

Amrein [123]	2011	Austria	Single-center multidisciplinary ICU patients	25	ELISA	Single dose of cholecalciferol 540,000 IU	Oral	No statistical difference in assessed clinical outcome including mechanical ventilation, vasopressor dependency, hospital stay, ICU stay, and hospital mortality	None
Amrein [93]	2014	Austria	Single-center multidisciplinary ICU patients	475	LC-MS/MS	Single dose of cholecalciferol 540,000 IU followed by a monthly dose of 90,000 IU for 5 months	Oral	No statistical difference in assessed clinical outcomes including hospital length of stay, hospital mortality, and 6-month mortality. Significant lower hospital mortality observed in critically severe vitamin D-deficient patients.	At month 6, 4 of 37 vitamin D-supplemented patients (11%) developed total calcium levels of > 10.6 mg/dL vs 1 of 43 patients in placebo group
Leaf [124]	2014	USA	Multicenter multidisciplinary ICU patients with severe sepsis and septic shock	67	LC-MS/MS	Single dose of calcitriol 2 μg	Intravenous	No influence on clinical outcome, mixed effects on inflammatory markers	None
Quraishi [125]	2015	USA	Single-center multidisciplinary ICU patients with severe sepsis and septic shock	30	ELISA	Single dose of cholecalciferol 200,000 or 400,000 IU	Oral	Associated with increased cathelicidin antimicrobial peptide levels, no effect on c-reactive protein	None
Nair [126]	2015	Australia	Single-center multidisciplinary ICU patients with systemic inflammatory response syndrome	50	LC-MS and ELISA	Single dose of cholecalciferol 150,000 or 300,000 IU	Oral	Associated with increased levels of cathelicidin antimicrobial peptide and reduction in pro-inflammatory cytokine interleukin 6	None
Han [127]	2016	USA	Multicenter multidisciplinary ventilated ICU patients	30	IA and ELISA	Daily doses of cholecalciferol 50,000 or 100,000 IU for 5 days	Oral	Hospital length of stay significantly decreased	None
Alizadeh [128]	2016	Iran	Single-center surgical ICU patients	59	ELISA	Single dose of cholecalciferol 600,000 IU	Intramuscular	Adiponectin, biomarker of insulin sensitivity, is significantly elevated. No effect on homoeostasis model assessment for insulin resistance and homestasis model assessment adiponectin.	Not reported
Han [129]	2017	USA	Multicenter multidisciplinary ventilated ICU patients	30	ELISA	Daily doses of cholecalciferol 50,000 or 100,000 IU for 5 days	Oral	Associated with increasing systemic levels of mRNA expression of human cationic antimicrobial protein overtime. No effect on circulating cathelicidin and human β-defensin.	None

Rousseau et al. have shown improved muscle recovery and strength in burn patients supplemented with vitamin D and implied that vitamin D supplementation had positive effects on muscle health and may play a role during rehabilitation [83]. This is the only study investigating the therapeutic potential of vitamin D following thermal injury. This lack of interventional studies could be due to a variety of reasons. First, as detailed earlier in this review, interpretation of vitamin D status has proven difficult owing to fluid shifts and low protein levels occurring in the acute response. It is assumed that binding protein levels return to normal 1 year post-injury, and therefore, “true” vitamin D status can be analyzed and managed [96]. Second, vitamin D deficiency in burn patients is believed to be a long-term issue [96], requiring a lengthy trial intervention. Third, there is still considerable debate about what vitamin D dosing regimen is optimal for supplementation studies as therapeutic benefit in relation to target serum concentrations of vitamin D remains unclear. In a recent meta-analysis of the effects of vitamin D supplementation on acute upper respiratory infection, positive response to vitamin D supplementation was only observed in patients receiving lower dose daily or weekly supplementation rather than monthly high-dose boluses of vitamin D [97].

### Current knowledge gaps and limitations to progress

Clinical trials investigating the effects of vitamin D supplementation on vitamin D status and outcomes in burn and other critically ill patients are scarce. Correction of vitamin D depletion in burn patients may prove beneficial, and further studies investigating the therapeutic potential of vitamin D should be encouraged.

A major limitation of this type of study is the different techniques used to analyze vitamin D levels in patients and the specific vitamin D metabolites measured in each instance. Methods used to quantify the most commonly analyzed vitamin D metabolite—25(OH)D—include immunoassay (IA), high-performance chromatography (HPLC) or liquid chromatography tandem–mass spectrometry (LC-MS/MS). IA measurements are subject to matrix effects, such as hemodilution, and therefore may not be as accurate [98]. Additionally, several IA methods cannot distinguish between the two main forms of vitamin D (vitamin D_3_ and vitamin D_2_), and cross-reactivity of vitamin D_3_ and other vitamin D metabolites can occur [99]. Vitamin D measurements by IA can also be influenced by VDBP concentration, thereby reducing its reliability [100]. LC-MS/MS involves chromatographic separation and mass transition differences of molecules allowing the differentiation of analytes, thereby increasing selectivity and sensitivity.

Due to differences in the sensitivity of each technique, the data generated may vary in accuracy and reproducibility. Therefore, analyzing and interpreting data obtained using different methodologies in various studies may not be possible. With this in mind, LC-MS/MS has proven to be the only platform offering an accurate, reliable, and reproducible method for quantification of serum 25(OH)D [99], including studies involving critically ill patients [101]. However, as outlined above, several other vitamin D metabolites, notably active 1,25(OH)_2_D, are likely to play a key role in the physiological activity of vitamin D in critically ill patients. Thus, new analytical strategies are required that incorporate simultaneous measurement of several vitamin D metabolites. LC-MS/MS protocols have been developed allowing accurate simultaneous analysis of various vitamin D metabolites in serum [102]. In addition, new protocols have been developed for the measurement of “free” (VDBP-unbound) 25(OH)D [103]. This bioavailable form of 25(OH)D is thought to play a crucial role in mediating immunomodulatory responses [104]. In future studies of vitamin D and critical illness, it will be important to incorporate all these new technologies.

## Conclusion

Thermal injury and critical illness influence vitamin D levels. Although data are scarce, vitamin D is most frequently found to be deficient in these patients and may potentially influence short- and long-term outcomes in burn patients and the critically ill. Further studies investigating vitamin D depletion in burn patients and its influence on prognosis, via standardized methodology such as LC-MS/MS, are necessary to reach definitive conclusions and influence clinical practice. Although two major RCT trials, VIOLET (NCT03096314) and VITdAL-PICU (NCT02452762), investigating vitamin D supplementation are active, recruited patients will only be given a one-off high dose of vitamin D. More RCTs using “optimal” vitamin D regimens in well-stratified patients are required to determine the utility and risk-benefit ratio of supplementation. These studies are essential to stimulate further clinical and scientific research assessing vitamin D phenomenon in critical care settings, which would be pivotal in determining the need for change in clinical practice.

## Abbreviations

1,25(OH)_2_D: 1α,25-Dihydroxyvitamin D
25(OH)D: 25-Hydroxyvitamin D
7-DHC: 7-Dehydrocholesterol
CYP27B1: 25-Hydroxyvitamin D-1α-hydroxylase
GM-CSF: Granulocyte-macrophage colony-stimulating factor
HPLC: High-performance liquid chromatography
IA: Immunoassay
ICU: Intensive care unit
IL: Interleukin
LC-MS/MS: Liquid chromatography tandem–mass spectrometry
VDBP: Vitamin D-binding protein
